# Conditional knockout of C/EBPβ in epidermis results in dysregulated lipid biosynthesis and a defect in skin barrier function

**DOI:** 10.1371/journal.pone.0326670

**Published:** 2025-06-25

**Authors:** Kevin J. Mills, Michael Doyle, John S. House, John G. Witherspoon, Daniel Krakko, Whitney L. Stutts, Jessie R. Chappel, Jonathan R. Hall, Erin S. Baker, Robert C. Smart

**Affiliations:** 1 Department of Biological Sciences, North Carolina State University, Raleigh, North Carolina, United States of America; 2 Department of Chemistry, North Carolina State University, Raleigh, North Carolina, United States of America; 3 Graduate Program in Toxicology, North Carolina State University, Raleigh, North Carolina, United States of America; 4 Center for Human Health and the Environment, North Carolina State University, Raleigh, North Carolina, United States of America; 5 National Institute of Environmental Health Sciences, Research Triangle Park, Durham, North Carolina, United States of America; 6 Molecular Education, Technology and Research Innovation Center (METRIC), North Carolina State University, Raleigh, North Carolina, United States of America; 7 Department of Molecular and Structural Biochemistry, North Carolina State University, Raleigh, North Carolina, United States of America; 8 Bioinformatics Research Center, Department of Biological Sciences, North Carolina State University, Raleigh, North Carolina, United States of America; 9 Department of Chemistry, University of North Carolina, Chapel Hill, North Carolina, United States of America; South China Agricultural University, CHINA

## Abstract

CCAAT/enhancer binding protein-β (C/EBPβ) is a basic leucine zipper transcription factor that is abundantly expressed in epidermal keratinocytes of skin. In the present study, C/EBPβ epidermal specific conditional knockout (CKOβ) SKH1 mice were utilized to interrogate C/EBPβ’s role in lipid biosynthesis and skin barrier integrity. RNAseq data analysis and gene set enrichment analysis of RNA isolated from the epidermis of CKOβ and K5Cre control mice revealed that deletion of C/EBPβ in epidermis resulted in an enrichment of downregulated genes in gene sets associated with lipid metabolism. Further analysis showed the majority of differentially regulated genes were downregulated in gene sets related to the metabolism/biosynthesis of ceramides, fatty acids, phospholipids, sphingolipids, and cholesterol species in CKOβ epidermis. Ingenuity Pathway Analysis predicted inhibition of multiple pathways involving lipid biosynthesis. Lipidomic analysis of epidermis using advanced chemical separations and tandem mass spectrometry identified 470 individual lipids in epidermis with 165 significantly decreased and 82 significantly increased in CKOβ epidermis. The lysophospholipids were the most decreased class of lipids, and free fatty acids and ceramides important in barrier formation were also decreased. The sphingomyelin class of lipids was the most increased. High resolution mass spectrometry for cholesterol lipids revealed several cholesterol esters were also dysregulated in CKOβ epidermis. Finally, we assessed the functional consequences of the loss C/EBPβ on epidermal barrier function and found that basal permeability barrier function as measured by transepidermal water loss (TEWL) was impaired, with an approximate doubling of TEWL in CKOβ mice. These results indicate that C/EBPβ is a is a major regulator of the epidermal lipidome and the deletion of C/EBPβ in epidermis leads to a defect in skin barrier function.

## Introduction

CCAAT/enhancer binding proteins (C/EBPs) are members of the basic leucine zipper class of transcription factors and act as homo- or heterodimers [1, 2] to orchestrate tissue-specific gene expression in response to hormones, growth factors, cytokines and nutrients [3, 4]. Depending upon the cell type, these transcription factors have important roles in proliferation, apoptosis, inflammation, senescence, differentiation and energy metabolism. C/EBPα and C/EBPβ are abundantly expressed in the epidermis [5, 6]. C/EBPβ is predominantly expressed in the suprabasal layers of the epidermis [5] and is activated during calcium-induced differentiation of keratinocytes in culture. C/EBPβ knockout mice display consistent significant changes, albeit modest, in the epidermis including decreased expression of keratin 1 and 10 and increased basal cell proliferation [7, 8]. C/EBPα is dispensable for epidermal homeostasis and yet the co-ablation of C/EBPα and C/EBPβ in mouse epidermis revealed that C/EBPα and C/EBPβ are essential for sebocyte differentiation and stratified squamous differentiation [9]. In addition, deficiency of C/EBPβ in epidermal cells revealed complex roles in skin carcinogenesis, keratinocyte and skin tumor cell survival, and innate immunity [9–12]. Collectively, these data suggest an important role for C/EBPs, especially C/EBPβ in regulating gene expression cascades that drive cell survival and differentiation in the skin.

Both C/EBPα and C/EBPβ have been shown to be important regulators of lipid metabolism in a variety of tissues and cell types [13–16], but to our knowledge, this has not been investigated in the epidermis. Because lipid metabolism in the epidermis is so inextricably linked to proper differentiation and the establishment and maintenance of competent permeability barrier function [17, 18], we utilized a C/EBPβ epidermal conditional knockout mouse model (CKOβ) to interrogate specifically how the loss of C/EBPβ affected the lipid metabolic gene expression profile, and whether gene expression changes were accompanied by changes in epidermal lipids and skin barrier function. In this study we focused on C/EBPβ rather than C/EBPα because we have in hand a tractable model, the CKOβ mouse, with which we recently completed epidermal RNAseq studies [12]. The present study demonstrates C/EBPβ plays a key role in the regulation of epidermal lipid gene expression pathways in SKH1 mice. The deletion of C/EBPβ in the epidermis resulted in significant changes in epidermal lipid content with over half of the 470 lipid species measured in epidermis significantly changed including decreases in key skin barrier lipids, such as free fatty acids, ceramides and cholesterol esters. Mice lacking C/EBPβ expression in the epidermis also display hallmarks of disrupted epidermal homeostasis, including epidermal thickening, compromised skin barrier function and dysregulation of many genes involved in the establishment and maintenance of epidermal homeostasis.

## Materials and methods

### Mice

Conditional epidermal knockout mice; ${\text{K5Cre}}^{+/tg};C/ebp\beta^{fl/fl}$ (CKOβ) were obtained by crossing ${\text{K5Cre}}^{+/tg}$ (K5Cre) mice with $\text{C/ebp}\beta^{fl/fl}$ mice [19]. K5Cre mice were a gift from Angel Ramirez and Jose Jorcano [20]. All genotypes were backcrossed to SKH1 hairless female mice (Charles River Labs) for at least five generations [21]. All aspects of animal care and experimentation described in this study were conducted according to the NIH guidelines and were approved by the NC State University Institutional Animal Care and Use Committee (IACUC).

### Immunoblot analysis

SKH1 mice (8-12 weeks of age) were euthanized, and dorsal skin was removed and subjected to 10 s heat shock in 60^∘^C ${\text{diH}}_{2}\text{O}$ followed by 15 s in ice water [21]. Epidermis was easily scraped from dermis with a weighing spatula and collected. Epidermal protein lysates were prepared using RIPA buffer (1% NP-40, 0.5% sodium deoxycholate, 0.1% sodium dodecyl sulfate, 1 mM dithiothreitol, 1 mM sodium orthovanadate, 1 mM AEBSF, 1 × protease inhibitor cocktail (Roche) and 1× Halt phosphatase inhibitor cocktail (ThermoFisher) in PBS). SDS-PAGE and immunoblot analysis was conducted using antibodies against C/EBPβ (ab32358, Abcam), and β-actin (sc-8432, Santa Cruz). Antibodies were diluted (C/EBPβ 1:1000; β-actin 1:10,000) in TBS containing 1% BSA and 0.1% Tween20. Membranes were imaged using an AI680RGB camera imager (General Electric).

### Tissue staining

SKH1 mice (8-12 weeks of age) were euthanized and whole dorsal skin was laid flat on card stock, fixed in 10% phosphate-buffered formalin and then transferred to 70% ethanol after 18 hours. Tissues were embedded in paraffin using standard processing settings and then sectioned at 5 μm and placed onto charged slides. Slides were stained with routine hematoxylin and eosin (H&E) stains. Morphometric microscopic analysis was done using Nikon NIS Elements. Unstained mouse skin sections (5 μm) were deparaffinized and peroxidases were inactivated with 3% ${\text{H}}_{2}{\text{O}}_{2}$ and subjected to antigen retrieval using a 2100 retriever (Aptum) with citrate buffer (pH 6). Sections were treated with 3% ${\text{H}}_{2}{\text{O}}_{2}$ once more before being blocked with normal serum (goat or horse) before incubation overnight at 4^∘^C with the C/EBPβ antibody (Santa-Cruz, sc-150, 1:4000) Staining was visualized using species appropriate secondary antibodies from Vectastain Elite ABC kits (Vector Labs, mouse:PK-6102, rabbit:PK-6101) and DAB Peroxidase Substrate Kit (Vector Labs, SK-4100). Sections were then counterstained with hematoxylin. Positively stained cells were scored in the interfollicular epidermis. IHC staining controls include no primary antibody control and CKOβ skin as negative control for C/EBPβ staining.

### RNA isolation, RNAseq analysis and bioinformatic analysis

Four K5Cre and 3 CKOβ SKH-1 mice 8-12 weeks old, ~1:1 male:female ratio, were euthanized and total RNA was extracted from epidermis collected from whole skin via heat shock [21]. Epidermis was placed in QIAzol Lysis Reagent (QIAGEN Cat. No. 79306) and RNA was purified using a Zymo Scientific silica-based spin column (Zymo Research Cat. Nom. R1018) and treated with DNase 1 (Zymo Research Cat. No. E1010). Illumina RNA library construction and sequencing (25 M 75 bp paired-end reads/sample) was conducted by Systems Technologies Core at NC State’s Center for Human Health and the Environment. An average of ~28.5 million paired-end raw RNAseq data were generated for each replicate. The quality of raw sequence data was assessed using FastQC and the first 12 poor-quality bases were trimmed based on the quality matrix from the FastQC application. The remaining good-quality reads were aligned to the mouse reference genome (mm38 version 87) using STAR aligner [22]. Per-gene counts of uniquely mapped reads for each replicate sample were calculated using the htseq-count script from the HTSeq python package. Genes with numerous aliases were removed and represented as a single gene in the data. The count matrix was imported and normalized for sequence depth and distortion, and dispersion was estimated using DESeq2 Bioconductor package in the R statistical computing environment [23]. Differentially expressed genes were identified after applying multiple testing correction using the Benjamini–Hochberg (BH) procedure (false discovery rate (FDR) < 0.05) [24]. Gene set enrichment analysis (GSEA) was conducted using the Molecular Signature Database (MsigDB) [25, 26]. RNAseq data from K5Cre and CKOβ mice were also analyzed using Ingenuity Pathway Analysis (IPA; QIAGEN) to identify canonical pathways, upstream regulators and associated functions related to loss of C/EBPβ in epidermis. Data was analyzed using Right-tailed Fisher Exact Test with BH multiple hypothesis testing-corrected p-value. Data were filtered by BH adjusted p-value ≤ 0.05 and an absolute z-score of 2.

### Lipid extraction from epidermal tissue for LC-IMS-CID-MS analysis

Nineteen K5Cre SKH1 mice and 17 CKOβ SKH1 mice (8-12 weeks of age, ~1:1 male female) were euthanized and the epidermis collected from whole skin via heat shock [11]. Epidermal samples were weighed and lipids isolated from each epidermal sample using a modified Folch extraction [27, 28]. Epidermis was combined with 750 μL of –20^∘^C methanol and homogenized in 2.0 ml, 2.4 mm tungsten-carbide bead tubes for 5 min with a Fisherbrand 24 bead mill. Samples were transferred to Fisherbrand glass culture tubes containing 750 μL of -20^∘^C methanol where 3 mL of chloroform and 200 μL of water were added. The samples were then vortexed for 30 s, sonicated for 30 min, vortexed again for 30 s, and incubated for 1 h at 4^∘^C. Following incubation, 1.2 mL of water was added, and samples were centrifuged for 10 min at 1000 × g. A 300 μL aliquot of the bottom lipid layer was transferred to a Sorenson microcentrifuge tube and dried *in vacuo*. The dried lipids were reconstituted in 190 μL of –20^∘^C methanol and 10 μL of chloroform and then stored at –20^∘^C until analysis with liquid chromatography, ion mobility spectrometry, collision induced dissociation and mass spectrometry (LC-IMS-CID-MS).

### LC-IMS-CID-MS analysis

The epidermal lipid extracts were evaluated using an Agilent 1290 UPLC coupled to an Agilent 6560 IM-QTOF platform with a commercial gas kit and MKS Instruments precision flow controller. Each extract was evaluated with reverse phase liquid chromatography (RPLC) by injecting 10 μL onto a Waters CSH column (3.0 mm × 150 mm × 1.7 μm particle size). A 34-minute gradient with a flow rate of 250 μL/min as used for all analyses where Mobile Phase A (MPA) consisted of 10 mM ammonium acetate in 40:60 LC-MS grade acetonitrile/water and Mobile Phase B (MPB) was 10 mM ammonium acetate in 90:10 LC-MS grade isopropanol/acetonitrile. Both positive and negative mode electrospray ionization (ESI) analyses were performed on all samples to obtain as many identifications as possible. Following ESI, the ions were analyzed using the Agilent 6560 IM-QTOF MS platform [29, 30]. Collision cross sections (CCS) values were collected for all lipids detected and collision induced dissociation (CID) was performed with high purity nitrogen by ramping collision energies based on the ion arrival times analogous to previous IMS experiments [31, 32]. Alternating scans of no fragmentation and all-ions data independent acquisition (DIA) were used to collect precursor and fragmentation information at 1 sec/spectra for a mass range of 50-1700 *m/z*.

### Lipid annotations

Lipidomic spectra were annotated in Skyline by matching features to our in-house lipid library of 778 lipids containing LC, IMS, MS and MS/MS information [33–35]. Lipid annotations were made based on mass errors for all precursor annotations < 2 ppm and fragment mass error < 10 ppm to library values, LC retention times were within 2 s of predicted elution times derived from Skyline’s iRT feature and experimental CCS values within 1%. While the LC-IMS-CID-MS platform enables lipid fatty acyl and head group annotation, fatty acyl back bone attachment to the *sn-1* or *sn-2* positions, and double bond orientations and placement were not differentiable with this method [36]. Lipids were annotated with “_” to denote ambiguous fatty acyl positions and “/” when stereochemistry is known (e.g., PC(0:0_18:0) *versus* PC(0:0/18:0) or PC(18:0/0:0)). Lipids were also annotated with “a” or “b” to denote potential isomers. Additionally, features with multiple potential lipid matches are noted with “;” (e.g., PC(18:1_16:0); PC (18:0_16:1)). Lipids were also assigned annotations for their summed carbon and double bond number when individual fatty acyl information could not be obtained or annotated (e.g., PE(34:1)). All odd chain species were validated with standards for CID fragmentation.

Following identification, peak areas of all manually validated lipids were exported from Skyline.

### Statistical analysis and interpretation

For statistical analysis and interpretation, exported Skyline peak areas were transformed to a $\log_{2}$ scale and normalized against their total ion current (TIC) since tissue variability was possible. Samples were screened for potential outliers through analysis of the PAV-RMD algorithm [37], Pearson’s correlation and principal component analysis using the *pmartR* [38] package in R (Version 4.0.3) [39]. ANOVA tests performed using *pmartR* compared mean lipid peak areas of K5Cre mice to the CKOβ mice after adjusting lipid means to minimize effects of sex and age. Lipids having a p ≤ 0.05 were considered statistically significant. Lipids were visualized using the SCOPE cheminformatics toolbox [40–46]. The resulting SCOPE dendrograms enabled visualization of the comparisons between the CKOβ and K5Cre mice.

### Analysis of cholesterol lipids

Cholesterol lipids were isolated from epidermis using a modified Folch extraction [47]. All solvents were purchased from Fisher Scientific and were LC/MS grade except for HPLC grade chloroform. Epidermal samples from the 19 K5Cre and 17 CKOβ mice (same mice described above for LC-IMS-CID-MS) were removed from -80 ^∘^C freezer, placed on ice, and approximately 20 mg weighed into low-binding microcentrifuge tubes (Sorensen Bioscience). Internal standards were added to each sample, including 10 μL Avanti SPLASH LIPIDOMIX, 10 μL 100 mg/L cholesterol-d7 and 10 μL 200 mg/L cholesterol-3-sulfate-d7 (Avanti Polar Lipids, Inc.) as well as 10 μL 200 mg/L cholesterol-3-sulfate-d7 (Cambridge Isotope Laboratories). Lipid extraction was performed by adding 200 μL chloroform, 100 μL methanol and 75 μL water, then the samples were vortexed and homogenized using a 2010 Geno/Grinder (SPEX SamplePrep). Sample extracts were transferred to glass vials, microcentrifuge tubes were washed with 600 μL of 2:1 chloroform:methanol and the wash was pooled with the extracts. The extracts were vortexed, sonicated for 10 min in an ice bath, then 150 μL water was added and samples were centrifuged for 10 min at 1000 × g at 4 ^∘^C. The lower organic phase was transferred to a new tube and the aqueous layer was re-extracted with 600 μL chloroform and the chloroform fractions were combined. Samples were dried in a vacuum concentrator and reconstituted in 10 μL chloroform followed by 190 μL methanol.

### UHPLC-MS conditions

The analysis was performed using a Vanquish UHPLC instrument (Thermo Fisher Scientific) coupled to a Orbitrap ID-X Tribrid mass spectrometer (Thermo Fisher Scientific). Chromatographic separation was achieved on a Waters CSH C18 (100 × 2.1 mm, 1.7 μm particle size) column maintained at 50 ^∘^C. Mobile phase A was 10 mM ammonium formate and 0.1% (v/v) formic acid in 60:40 (v/v) acetonitrile:water, mobile phase B was 10 mM ammonium formate and 0.1% (v/v) formic acid in 90:10 2-propanol:acetonitrile. Mobile phases were sonicated for 30 min before use. The following linear gradient (with a flow rate of 0.4 mL/min) was used: 0 min (40% B), 2.0 min (43% B), 2.1 min (50% B), 12.0 min (54% B), 12.1 min (70% B), 18.0 min (99% B), 20 min (99% B), 20.1 min (40% B), 22.0 min (40% B).

The source parameters of the mass spectrometer were ion voltage: 3000 V, sheath gas: 60 (arb), aux gas: 20 (arb), sweep gas: 1 (arb), ion transfer tube temperature: 285 ^∘^C, vaporizer temperature: 320 ^∘^C. Samples were analyzed (2 μL injections) in positive and negative ionization mode separately with a scan range of *m/z* 150–1500. MS1 and MS2 resolving power was 120,000 and 15,000 (FWHM at *m/z* 200), respectively. The following ions were targeted for MS2: $[{\text{M-H}}_{2}\text{O}+\text{H}{]}^{+}$ for cholesterol; $[\text{M}+{\text{NH}}_{4}{]}^{+}$ for cholesterol esters; $[\text{M-H}{]}^{-}$ for cholesterol sulfate. Data-dependent MS2 scans were performed with a cycle time of 0.9 s, using stepped, normalized collision energies of 25, 30, 35% (NCE).

Cholesterol esters were identified using LipidSearch and Compound Discoverer (Thermo Fisher Scientific), then quantified in Skyline Daily. Cholesterol, cholesterol sulfate and CE(18:1) were quantified using internal standardization with external calibration. All other identified cholesterol esters were quantified using CE(18:1) as a surrogate. Seven calibration points were prepared in methanol in the range of 0.1 – 50 μg/mL.

### Measurement of barrier function by TEWL

Four K5Cre and 8 CKOβ SKH1 mice were anesthetized with 0.5ml (1ml initial charge) isoflurane in a bell jar using gauze doused for 45 seconds until complete loss of consciousness. Mice were removed from the bell jar and two ventral measurements were taken in succession immediately posterior to the sternum using a GPSkin Barrier Pro 1 instrument [48] for transepidermal water loss (TEWL) (${\text{g/m}}^{2}/\text{hr})$. The instrument’s chamber was aired and wiped out between measurements. The mice were monitored as they regained consciousness (~1 minute) and mobility.

## Results

### Conditional deletion of C/EBPβ in SKH1 mouse epidermis

**Fig 1:**
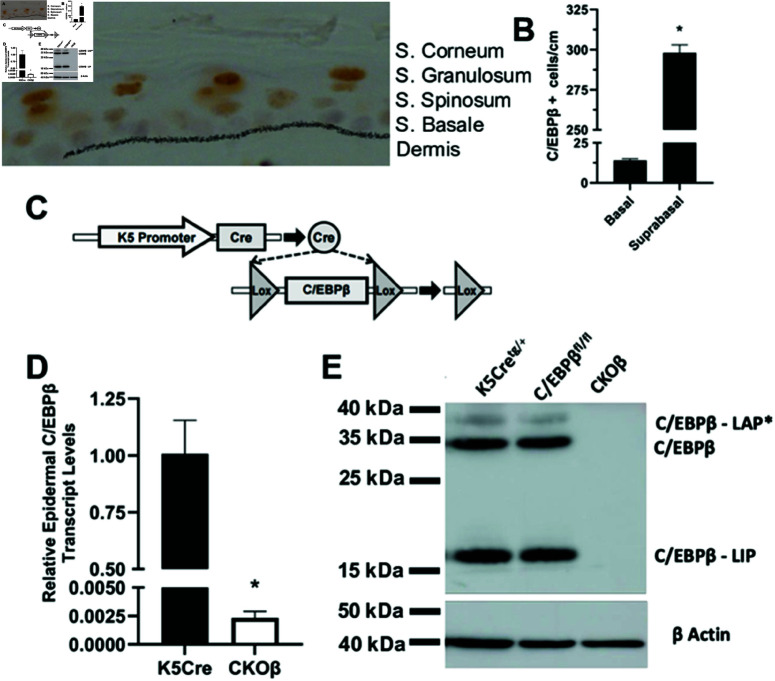
Localization and conditional deletion of C/EBPβ in SKH1 mouse epidermis. (A) IHC localization of C/EBPβ in SKH1 mouse epidermis; stippled black line represents the basement membrane separating the epidermis (above) from dermis (below). (B) Quantitation of C/EBPβ IHC positive cells in basal and suprabasal layers N=4 mice, data are expressed as the mean ± SD, * p< 0.05 Student’s t-test. (C) Schematic diagram showing genetic deletion of C/EBPβ in the epidermis of a K5Cre; $\text{C/EBP}\beta^{\text{fl/fl}}$ mouse. (D) Relative transcript levels in K5Cre and CKOβ mouse epidermis using TaqMan real-time PCR. N=3 mice/genotype, data are expressed as the mean ± SD, * p< 0.05 Student’s t-test. (E) Western blot analysis for C/EBPβ in epidermal homogenates from K5Cre, $\text{C/EBP}\beta^{\text{fl/fl}}$ and CKOβ mice. All three isoforms of C/EBPβ (C/EBPβ-LAP*, C/EBPβ and C/EBPβ-LIP) are detected in K5Cre and $\text{C/EBP}\beta^{\text{fl/fl}}$ mice but not in CKOβ mice. All 3 isoforms of C/EBPβ are produced from different translational start sites in C/EBPβ mRNA.

IHC staining for C/EBPβ in SKH1 epidermis shows C/EBPβ is localized to the nucleus of epidermal keratinocytes and its expression increases as basal epidermal keratinocytes move upward from the basement membrane into the suprabasal layers to undergo stratified squamous differentiation (Fig 1A). Highest C/EBPβ expression is in the stratum granulosum, intermediate expression in the stratum spinosum and lowest expression is in the stratum basale. Overall, 96% of the C/EBPβ positive stained keratinocytes were localized to suprabasal keratinocytes with only 4% localized to the basal keratinocytes (Fig 1B). To better understand the role of C/EBPβ in epidermal biology, we developed an epidermal conditional knockout of C/EBPβ (CKOβ) on the SKH1 hairless mouse background using Cre-Lox recombination (Fig 1C). The SKH1 mouse is hairless, immunocompetent, unpigmented and widely used in wound healing, photobiologic responses, skin cancer and dermatologic research [49]. In the CKOβ model, the keratin 5 (K5) promoter directs Cre recombinase expression to the epidermis to delete the floxed gene, $\text{C/EBP}\beta^{\text{fl/fl}}$ (Fig 1C) [12]. The deletion of C/EBPβ was confirmed by TaqMAN Real-Time PCR analysis of C/EBPβ transcripts in RNA isolated from K5Cre and CKOβ epidermis (Fig 1D) and by immunoblot analysis for C/EBPβ in lysates prepared from the Cre and CKOβ epidermis (Fig 1E). All three C/EBPβ protein isoforms were detected in the epidermis of K5Cre mice and as expected none were detected in CKOβ epidermis as the three isoforms of C/EBPβ are produced from different translational start sites on the C/EBPβ mRNA transcript.

### Deletion of C/EBPβ in epidermis results in down regulation of pathways and upstream regulators involved in epidermal lipid metabolism

**Fig 2:**
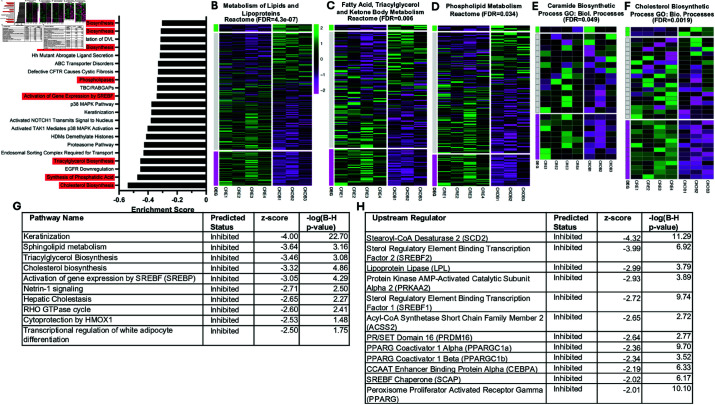
Conditional deletion of C/EBPβ in epidermis results in downregulation of transcripts and pathways associated with lipid biosynthesis in epidermis. (A) Analysis of epidermal RNAseq results from CKOβ and K5Cre mice using gene set enrichment analysis (GSEA) and the mouse Molecular Signature Database (MSigDB). Top 21 pathways with the most negative enrichment scores are shown. Highlighted gene sets are those associated with lipid metabolism. (B-F) Row scaled heatmaps of RNAseq data from CKOβ epidermis compared to K5Cre epidermis. Each column represents RNAseq results from epidermal RNA isolated from a single mouse. Overall FDR for each gene ontology grouping comparing CKOβ to K5Cre is shown at the top of each heat map group. All individual genes below the bottom white line are significantly downregulated in CKOβ epidermis and all genes above top white line are significantly upregulated in CKOβ epidermis FDR< 0.1. (G) IPA pathway analysis of RNAseq data reveals the top pathways predicted to be inhibited (z-score ≤ –2.0 are predicted to be inhibited) in epidermis of CKOβ mice compared to K5Cre mice. Right tailed Fisher Exact Test with Benjamini-Hochberg (B-H) multiple hypothesis testing-corrected p-value. (H) IPA’s upstream regulator analysis of RNAseq data reveals top upstream transcription regulators predicted to be inhibited (z-scores ≤ –2.0) based on observed gene expression changes in epidermis of CKOβ mice compared to K5Cre mice. Right tailed Fisher Exact Test with Benjamini-Hochberg (B-H) multiple hypothesis testing-corrected p-value.

We reported previously that the conditional deletion of epidermal C/EBPβ had a dramatic effect on the transcriptome of CKOβ mice when compared with K5Cre controls [12]. Transcriptomic analysis using RNAseq showed 2586 differentially expressed genes, with 1202 upregulated and 1384 downregulated genes (adj. p value ≤ 0.1) out of a dataset of 20,400 genes with the IFN pathway being the most highly enriched pathway among significantly upregulated genes in CKOβ epidermis [12]. In the present work, we analyzed the RNAseq results from the epidermal RNA isolated from CKOβ and K5Cre mice using GSEA and the MSigDB. GSEA revealed that deletion of C/EBPβ in epidermis resulted in negative enrichment scores (enrichment of downregulated genes in gene sets) for numerous gene sets associated with lipid metabolism (Fig 2A). In fact, 8 out of the top 21 data sets with the highest negative enrichment scores were associated with lipid metabolism (FDR < 0.05) (Fig 2A). Row scaled heat maps from Reactome and GO lipid-related data sets show significant differences (FDR < 0.05) between Cre and CKOβ mice with an enrichment of downregulated transcripts in CKOβ mouse epidermis (Figs 2B–2F; S1–S5 Tables). Of the top 10 pathways predicted to be inhibited from the IPA analysis of the dataset, six are associated with lipid metabolism (Fig 2G). IPA predicted numerous upstream regulators that best explained the observed changes in gene expression in the RNAseq data set and many were related to lipid homeostasis and were predicted to be inhibited (z score < –2.0) (Fig 2H) suggesting C/EBPβ could be directly or indirectly regulating other transcription factors that control lipid metabolism/biosynthesis. Finally, IPA predicted Dermatological Diseases and Conditions as a top Disease/Disorder impacted by the loss of C/EBPβ and dermatitis was predicted to be increased (z-score 2.55 B-H p-value 5.23E-19 and this was the only Dermatological Disease and Condition with a z-score above 2.0; none were below –2.0).

### Deletion of C/EBPβ results in a dramatic reduction in epidermal lipid content

**Fig 3:**
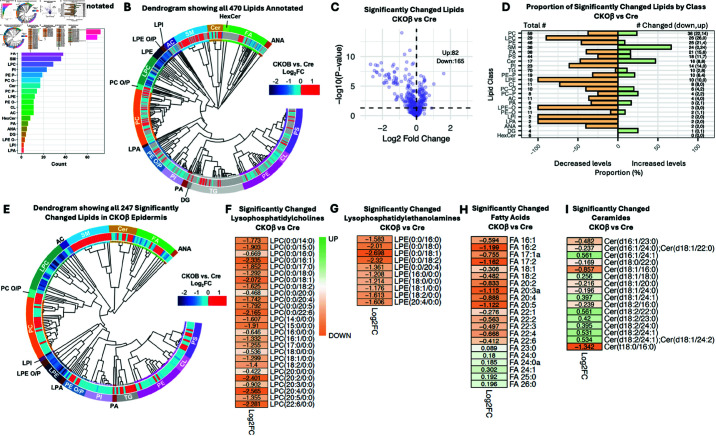
Lipidomic analysis of epidermis using LC-IMS-CID-MS reveals major changes in CKO/β/ epidermis. (A) Number of lipids annotated per lipid class. TG = Triacylglycerol, PC = Phosphatidylcholine, PE = Phosphatidylethanolamine, PS = Phosphatidylserine, FA = Fatty acid, SM = Sphingomyelin, LPC = Lysophosphatidylcholine, PI = Phosphatidylinositol, PE P = Alkenyl ether PE, PC O = Alkyl ether PC, Cer = Ceramide, PC P- = Alkenyl ether PC, LPE = Lysophosphatidylethanolamine, PE O = Alkyl ether PE, CL = Cardiolipin, AC = Acylcarnitine, HexCer = Hexosylceramide, PA = Phosphatidic acid, ANA = Anandamide, DG = Diacylglycerol, LPE O = Alkyl ether LPE, LPI = Lyso PI, LPA = Lyso PA. N=19 K5Cre mice and N=17 CKOβ mice. (B) Dendrogram showing all 470 lipids detected. Heatmap indicates direction of lipid mean $\log_{2}$ fold change on scales of red or blue for upregulated or downregulated lipids respectively for CKOβ vs K5Cre epidermis p≤0.05 ANOVA. Gray indicates lipids that are not significantly different in CKOβ vs K5Cre epidermis (p≤0.05 ANOVA). See (A) for lipid class nomenclature. (C) Volcano plot illustrates significantly changed lipids in CKOβ vs K5Cre epidermis, p ≤0.05 ANOVA, horizontal dashed line represents p≤ 0.05. (D) Proportion of significantly changed lipids by lipid class in CKOβ vs K5Cre epidermis. The total number of lipids detected for each class of lipid is shown in the column of numbers on the lefthand side of the figure. On the righthand side of the figure, the column of numbers represents the total number of lipids changed for each class in CKOβ epidermis followed in parentheses by the number of decreased and increased lipids. The yellow bars represent the percent of lipids in given class that are decreased, and green bars represent the percent of lipids in each class that are increased. (p< 0.05 ANOVA). See (A) for lipid class nomenclature. (E) Dendrogram showing all 247 significantly changed lipids in CKOβ epidermis. Heatmap indicates direction of lipid mean $\log_{2}$ fold change on scales of red or blue for upregulated or downregulated lipids respectively for CKOβ vs K5Cre epidermis respectively (p≤0.05 ANOVA). (F) Significantly changed lysophosphatidylcholines (LPC) in CKOβ vs K5Cre epidermis (p≤0.05 ANOVA) heatmap with mean $\log_{2}$ fold change shown for each lysophosphatidylcholine. (G) Significantly changed lysophosphatidylethanolamines (LPE) in CKOβ vs K5Cre epidermis (p≤0.05 ANOVA) heatmap with mean $\log_{2}$ fold change shown for each lysophosphatidylethanolamine. (H) Significantly changed fatty acids (FA) in CKOβ vs K5Cre epidermis (p≤0.05 ANOVA) heatmap with mean $\log_{2}$ fold change shown for each fatty acid. (I) Significantly changed ceramides (Cer) in CKOβ vs K5Cre epidermis (p≤0.05 ANOVA) heatmap with mean $\log_{2}$ fold change shown for each ceramide.

To determine whether the pronounced dysregulation of gene expression associated with lipid metabolism was manifested at the level of lipid content in CKOβ epidermis, we employed an untargeted lipidomics approach with a platform combining liquid chromatography, ion mobility spectrometry, collision induced dissociation and mass spectrometry (LC-IMS-CID-MS). A total of 470 distinct lipids from 23 different classes of lipids were detected (S6 Table). The number of lipids annotated in each class is shown in Fig 3A with triglycerides and phosphatidylcholines having the most species identified, but also the most species in the in-house library. Additionally, the lipid species found in the epidermis using this method are illustrated in the dendrogram heat map (Fig 3B). The dendrogram specifically shows the classes of lipids and whether the individual species within each class were unchanged (gray) or significantly increased (red) or decreased (blue) in the epidermis of CKOβ mice compared to the epidermis of K5Cre mice. As illustrated in the Volcano plot (Fig 3C), a total of 247 lipids were statistically significantly different between CKOβ vs K5Cre epidermis, with 165 species decreasing, and 82 increasing in CKOβ epidermis. Interestingly, half of the lipids detected were significantly different in CKOβ epidermis and approximately 1/3 of all the lipids detected were significantly decreased in CKOβ epidermis. The proportion of significantly changed lipids is shown by class in Fig 3D and in the dendrogram heat map in Fig 3E. Specifically, all the significantly changed lysophosphatidylethanolamine and lysophosphatidylcholine species were decreased (Fig 3F/3G) and had the greatest fold change observed for all lipid classes with some species decreasing by 4-fold, shown in heatmaps as specific lipids with a ≤–2.0 ${\text{Log}}_{2}\text{FC}$. Additionally, all significant lipids in the phosphatidylinositol, anandamide, cardiolipin, and acylcarnitine classes decreased (S6 Table). In contrast, the sphingomyelin class was the only lipid class where all significant species increased (S6 Table). The significantly changed fatty acids (Fig 3H) and ceramides (Fig 3I) are shown in heat maps as lipids in these classes are important in skin barrier function. Collectively, the above results demonstrate that deletion of C/EBPβ in the epidermis leads to considerable changes in lipid content in the epidermis and indicate C/EBPβ is a major regulator of the epidermal lipidome.

### Deletion of C/EBPβ in the epidermis results in changes in cholesterol esters

**Fig 4:**
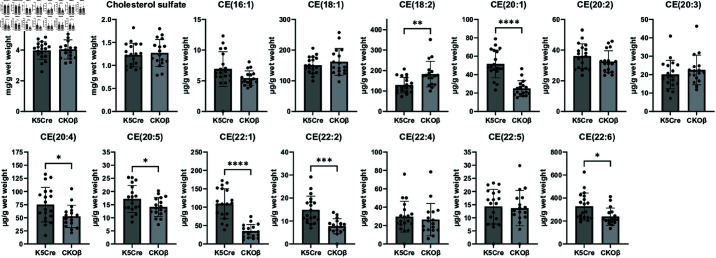
Targeted analysis of cholesterol and cholesterol esters in epidermis reveals changes in specific cholesterol esters in CKOβ epidermis. Cholesterol and cholesterol esters were measured in epidermis using UHPLC-MS. Bar graph represents the mean ± SD N=19 K5Cre mice and N=17 CKOβ mice. * p< 0.5, ** p< 0.01, *** p< 0.001, **** p< 0.0001 Student’s t-test.

Cholesterol and cholesteryl esters were quantitated by UHPLC-MS (Fig 4). Cholesterol and cholesterol sulfate levels were unchanged in CKOβ epidermis (Fig 4). Of the 13 cholesterol esters identified six cholesterol esters were significantly decreased in CKOβ epidermis and one significantly increased (Fig 4, S7 Table). Thus, deletion of C/EBPβ in the epidermis impacts the levels of cholesterol esters in the epidermis.

### Loss of epidermal C/EBPβ leads to a defect in skin basal permeability barrier function

**Fig 5:**
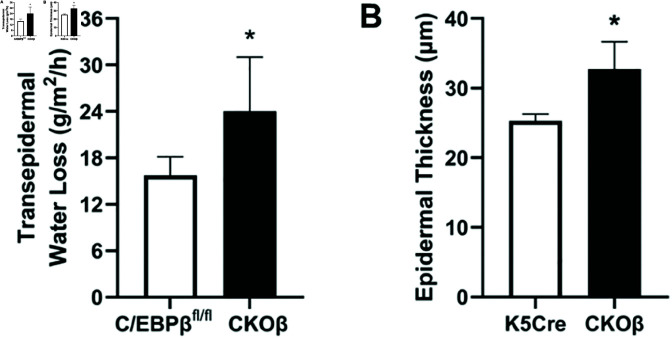
CKOβ mice display decreased skin barrier function and perturbed homeostasis. (A) Transepidermal water loss (TEWL) in $\text{C/EBP}\beta^{\text{fl/fl}}$ and CKOβ SKH1 mice. TEWL was measured with GPSkin Barrier Pro 1, N=8 $\text{C/EBP}\beta^{\text{fl/fl}}$ mice and N=4 CKOβ mice, data are expressed as the mean ± SD, * p < 0.05 Student’s t-test. (B) Epidermal thickness of K5Cre and CKOβ mice measured in H&E stained FFPE skin sections using Nikon NIS software. N=4 mice/group, data are expressed as mean ± SD p< 0.05 Student’s t-test.

We hypothesized that a functional consequence of the alterations in lipid metabolism resulting from the loss of C/EBPβ in the epidermis would be compromised permeability barrier function due to insufficient barrier lipid production/processing and/or altered ratios of barrier lipids in the stratum corneum lipid lamellae. We further hypothesized that compromised permeability barrier function would be accompanied by additional indicators of disrupted epidermal homeostasis, such as increased epidermal thickness, as epidermal thickening that results from hyperplasia can cause or contribute to diminished barrier function due to a disruption in the ordered processes that govern the establishment and maintenance of homeostasis in the skin. A salient example of this is psoriasis, a skin disease in which keratinocyte hyperproliferation, in concert with over-exuberant inflammatory signaling results in lesion formation and skin barrier dysregulation [50]. Permeability barrier function was assessed by measuring TEWL. CKOβ mice displayed significantly higher basal TEWL than $\text{C/EBP}\beta^{\text{fl/fl}}$ controls, indicative of an impairment in permeability barrier function (Fig 5A). The loss of C/EBPβ expression resulted in a 25% increase in epidermal thickness (Fig 5B), indicative of a disruption in the balance of cell proliferation and differentiation in the skin.

## Discussion

The purpose of this study was to characterize the effect of conditional deletion of epidermal C/EBPβ on the expression of genes involved in lipid metabolism, and to determine what such effects might have on epidermal lipid content, homeostasis and permeability barrier function. Examination of global epidermal gene expression using GSEA and IPA canonical pathway and upstream regulator analyses found highly significant changes in the expression of genes involved in epidermal lipid metabolism and skin barrier function. The *in-silico* analyses reported here support an important and previously unknown role for C/EBPβ as a major regulator of lipid metabolism and the lipidome in the epidermis, a highly active site of lipid production [51].

A particularly striking feature of the CKOβ transcriptome was down-regulation of many of the most important genes encoding proteins involved in the manufacture of epidermal lipids. This was accompanied by pronounced changes in the content of several classes of lipids important in epidermal signaling and permeability barrier function, including lysophospholipids, free fatty acids, ceramides, sphingomyelin, and phospholipids. It was also found that there was a slight thickening in the viable epidermis of CKOβ mice, indicating a disruption of the normal processes governing the balance of cell proliferation and differentiation in the skin. One observed functional consequence of loss of C/EBPβ in the epidermis was an impairment of epidermal permeability barrier function, where basal TEWL was significantly higher in CKOβ vs $\text{C/EBP}\beta^{\text{fl/fl}}$ cntrols. This is likely the result of the altered lipid content resulting in relative abundance changes in ceramides, cholesterol lipids and free fatty acids. Equimolar ratios of these lipids are important for optimal permeability barrier function and are often altered in skin diseases like psoriasis and atopic dermatitis [52–55]. Such changes in lipid composition can disrupt the highly ordered molecular interactions in the stratum corneum “brick and mortar” model wherein corneocytes (bricks) are embedded in a matrix of lipid (mortar), the optimal structure of which maintains several gradients and enzymatic activities in order to prevent water loss in a terrestrial environment and protect against physical and chemical insults [56, 57]. Perturbed barrier function is seen in virtually all skin diseases either as a primary trigger or a secondary consequence of disease pathogenesis. In keeping with the defect in basal barrier function, as well as the previously reported increase in the expression of genes involved in innate immunity [12], it was found that a number of genes involved in inflammation, wounding, hyaluronan metabolism and keratinization were significantly increased in CKOβ vs K5Cre epidermis. Upregulation of genes involved in inflammation and epidermal differentiation is a well-known homeostatic response to stratum corneum damage and is a feature of a wide range of skin conditions characterized by compromised barrier function [58, 59]. It was also found that several key genes e.g. filaggrin, loricrin, and corneodesmosin, which are involved in the establishment of competent epidermal barrier function were significantly decreased. Together, these results indicate that the loss of C/EBPβ expression leads to a disruption of epidermal homeostasis. These gene expression findings underlie the IPA prediction of Dermatological Diseases and Conditions as a top Disease/Disorder impacted by the loss of C/EBPβ and align well with other measures of skin barrier function and epidermal thickness.

Most of the epidermal lipid content findings follow straightforwardly from the gene expression profile. For example, FA < 24 carbons were significantly downregulated, as was the rate limiting enzyme in the biosynthesis of fatty acids, acetylCoA carboxylase alpha (*Acaca*). Similarly, important genes in the biosynthesis of CER, including serine palmitoyl transferase (*Sptlc1*), the rate limiting enzyme in sphingolipid biosynthesis, ceramide synthase 4 (*Cers4*), which catalyzes the formation of ceramide from sphinganine and acylCoA substrates, delta 4 desaturase sphingolipid 1 (*Degs1*), which catalyzes the oxidation of dihydroceramide to ceramide, and sphingomyelinase (*Smptd1*) were significantly downregulated in CKOβ epidermis (Fig 2) [12]. This latter finding may also be a contributing factor to the increased content of sphingomyelin found in CKOβ epidermis. Despite these findings, more than half of the ceramide species detected in CKOβ epidermis were unchanged or even upregulated (Fig 3). Consistent with this, *Cers2*, *Cers3*, *Cers5* and *Cers6*, all isoforms of ceramide synthase, were unchanged at the level of gene expression, as were UDP-glucose:ceramide glucosyltransferase (*Ugcg*) and β-glucocerebrosidase (*Gba*, *Gba2*) isoforms [12]. This indicates that while loss of C/EBPβ expression perturbed sphingolipid homeostasis even at the most proximal levels of the pathway, it was not a systematic, insuperable effect.

Other lipid classes that were significantly decreased in CKOβ epidermis were lysophospholipids (e.g. LPC, LPE, LPE-O and LPA) and some phospholipids (e.g. PI and PE). A possible explanation for the diminished content of lysophospholipids and hence, other phospholipids is that the genes encoding secreted phospholipases A2 (*Pla2G2e*, *Pla2g3* and *Pla2g7*) were significantly downregulated, as was membrane bound lipase H (*Liph*) in CKOβ epidermis. Perturbation of the expression of these phospholipases can affect epidermal homeostasis on more than one level. Not only are these enzymes necessary for many signal transduction pathways involving the release of fatty acids at the sn-2 position [60], but the larger family of phospholipases A2 is emerging as an increasingly important player in the biology of the skin [61–63]. Altered steady state levels of lysophospholipids can have additional implications for epidermal homeostasis, as it has been shown that lysophosphatidic acid can control the expression of filaggrin (flg), and has many other effects on physiological and pathological processes in the skin [64, 65]; indeed, flg was significantly downregulated in CKOβ epidermis [12].

Other lipid content findings in CKOβ epidermis may seem less readily explained by consulting the broad patterns of the gene expression profile. For example, there was a significant increase in the content of triacylglycerols and long chain fatty acids in CKOβ epidermis even though many of the enzymes involved in biosynthesis of both classes of lipids were downregulated. However, long chain fatty acids can either be synthesized in the epidermis, or enter the skin from extracutaneous sources, and it is at least plausible that some of our findings could be explained by an alteration in the expression of lipid transporters. Overall, our gene expression and lipid content findings may be due to the built-in redundancies of C/EBPα and C/EBPβ signaling [9] and/or to the stimulation of homeostatic compensatory mechanisms typically seen in the setting of compromised barrier function, and which have been documented in GEMMs before, especially those interrogating aspects of epidermal permeability barrier function [66, 67].

A lack of perfect concordance between gene and protein expression is well established [68, 69], and given that so many different levels of regulation may be in play, including post-transcriptional, post-translational, epigenetic modifications, feedback regulation, etc., the ultimate explanation for the phenotypic end product measurements in the CKOβ mice will require further research to unravel.

It should be noted that apart from dry flaky skin in older mice there is no macroscopically obvious cutaneous phenotype in the CKOβ mice. However, others have reported that systemic C/EBPβ knockout mice on a MF1:CBA:C57BL/6 strain background develop spontaneous skin lesions [70]. Genetic differences in the mouse strains used in these GEMMs and/or environmental conditions could account for the different skin phenotypes. Future studies will be aimed at examining whether challenging the skin barrier of CKOβ mice and control mice with pathogens and environmental toxicants will reveal an amplified adverse skin phenotype and whether percutaneous absorption in CKOβ mice is significantly altered.

In conclusion, we show that the loss of C/EBPβ causes a pronounced alteration in the expression of many genes involved in lipid metabolism in the epidermis. These changes are accompanied by clear changes in lipidome, hallmarks of disrupted epidermal homeostasis, and impaired permeability barrier function.

## Supporting information

S1 TableMetabolism of lipids and lipid proteins CKOβ vs K5Cre Reactome(CSV)

S2 TableFatty acids, triacylglycerol and ketone body metabolism CKOβ vs K5Cre Reactome.(CSV)

S3 TablePhospholipid metabolism CKOβ vs K5Cre Reactome.(CSV)

S4 TableCeramide biosynthetic process CKOβ vs K5Cre GO: Biological Processes.(CSV)

S5 TableCholesterol biosynthetic process CKOβ vs K5Cre GO: Biological Processes.(CSV)

S6 TableLC-IMS-CID-MS CKOβ vs K5Cre results.(CSV)

S7 TableUHPLC-MS CKOβ vs K5Cre results.(XLSX)

S1 Raw images Uncropped raw western blot images. (TIF)
