# Supplementary figures and images for: Conditional knockout of C/EBPβ in epidermis results in dysregulated lipid biosynthesis and a defect in skin barrier function
Source: PLoS One. 2025 Jun 25;20(6):e0326670. doi: 10.1371/journal.pone.0326670 (PMC12194017; doi:10.1371/journal.pone.0326670)

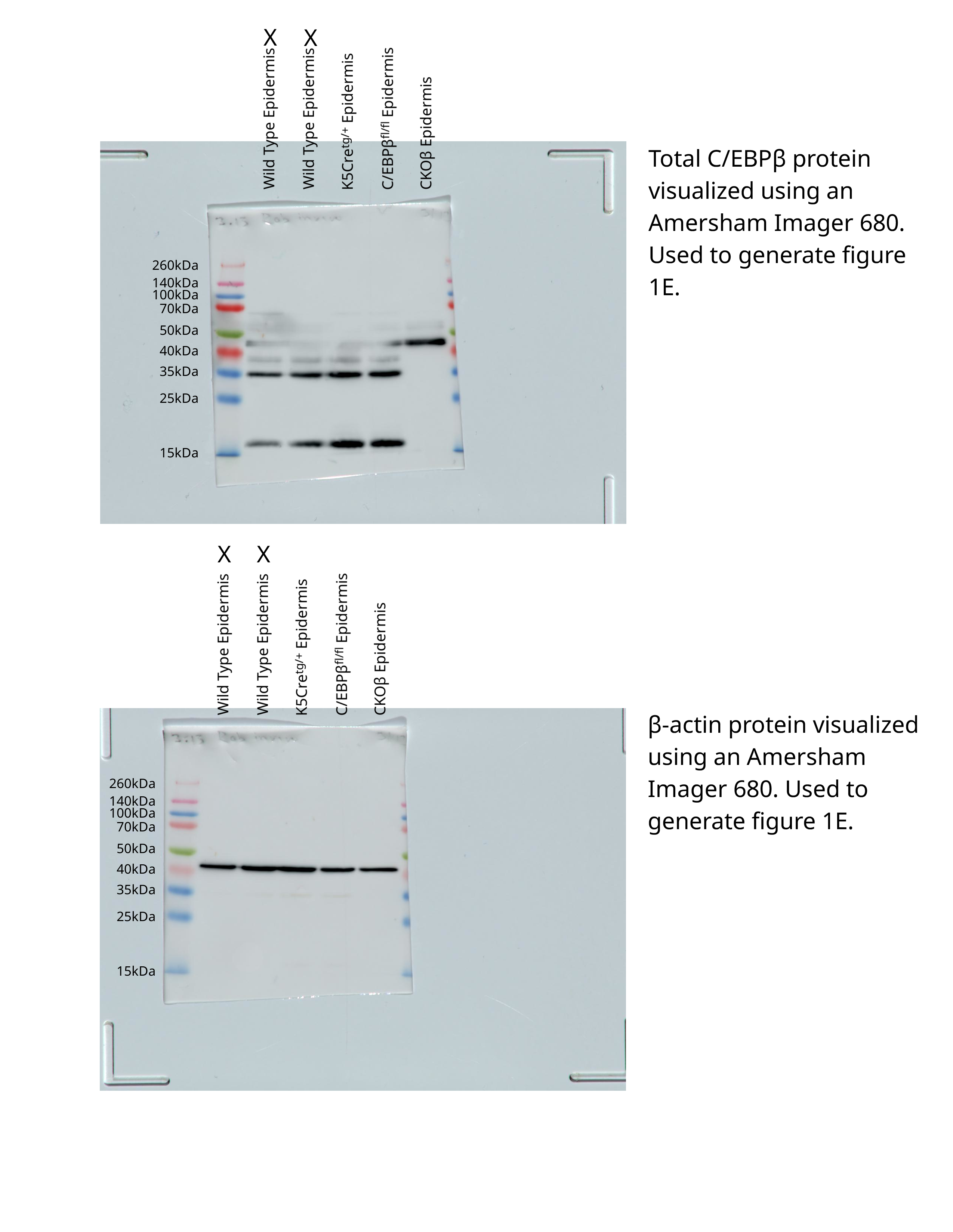

Supplement: S1 Raw images — (TIF) [file pone.0326670.s008.tif]
